# Twenty-four hours secretion pattern of serum estradiol in healthy prepubertal and pubertal boys as determined by a validated ultra-sensitive extraction RIA

**DOI:** 10.1186/1472-6823-8-10

**Published:** 2008-09-25

**Authors:** Carina Ankarberg-Lindgren, Ensio Norjavaara

**Affiliations:** 1 Göteborg Pediatric Growth Research Center, Department of Pediatrics, Institute of Clinical Sciences, The Sahlgrenska Academy at University of Gothenburg, S-41685 Göteborg, Sweden

## Abstract

**Background:**

The role of estrogens in male physiology has become evident. However, clinically useful normative data for estradiol secretion in boys has not previously been established due to the insensitivity of current methods used in clinical routine. By use of a validated ultra-sensitive extraction RIA, our aim was to establish normative data from a group consisting of healthy boys in prepuberty and during pubertal development.

**Methods:**

Sixty-two 24-hours serum profiles (6 samples/24 hours) were obtained from 44 healthy boys (ages; 7.2–18.6 years) during their pubertal development, classified into five stages: prepuberty (testis, 1–2 mL), early (testis, 3–6 mL), mid (testis, 8–12 mL), late-1 (testis,15–25 mL, not reached final height) and late-2 (testis,15–25 mL, reached final height). Serum estradiol was determined by an ultra- sensitive extraction radioimmunoassay with detection limit 4 pmol/L and functional sensitivity 6 pmol/L.

**Results:**

Mean estradiol concentrations during 24-hours secretion increased from prepuberty (median: <4 (5–95 percentiles: <4 – 7) pmol/L) to early puberty (6 (<4 – 12 pmol/L) but then remained relatively constant until a marked increase between mid-puberty (8 (4 – 17) pmol/L) and late-1 (21 (12 – 37) pmol/L) puberty, followed by a slower increase until late-2 puberty (32 (20 – 47) pmol/L). The diurnal rhythm of serum estradiol was non-measurable in pre- and early puberty, but discerned in mid-puberty, and become evident in late pubertal stages with peak values at 0600 to 1000 h.

**Conclusion:**

With the use of an ultra-sensitive extraction RIA, we have provided clinically useful normative data for estradiol secretion in boys.

## Background

During the last 10–15 years, the role played by estrogens in male physiology has been highlighted. Apart from normal physiology processes involving the reproductive system, growth spurt, epiphyseal closure and bone mineralization [1-3], estrogen has been suggested to have a role in pathological processes in male infertility in obesity [4] and cardiovascular diseases [5]. To study these conditions in boys and to be able to mimic physiological concentrations during estrogen, androgen or aromatase inhibitor therapy, we need information of both androgen and estradiol serum levels. So far androgen levels and diurnal variation in boys are well characterized [6-8], but normative data for the 24-hours secretion pattern of estradiol in boys throughout puberty is still lacking.

Determination of serum estradiol concentrations in boys has been fraught with problems of analytical insensitivity, lack of specificity [9] and has been done without validation or attention to the pitfalls when determining low serum estradiol concentrations, resulting in misleading reference intervals. Most commercial available immunoassays significantly overestimate estradiol concentrations at the low levels found in children, men and postmenopausal women, though results can be erroneously low as well [10-13]. Assays that utilize an organic solvent extraction step to remove interfering substances, such as non-specific-bindings and binding globulins, have been shown to give lower values which more resemble values measured by MS-based methods [12,13].

In the present study, we present data on the 24-hours secretion of serum estradiol in healthy boys throughout puberty in relation to their testosterone secretory dynamics, determined by a validated ultra-sensitive extraction estradiol RIA with detection limit 4 pmol/L and functional sensitivity 6 pmol/L.

## Methods

### Study group

The study group consisted of 44 healthy Swedish boys derived from a group described in a previous report [8]. Twenty-one of the boys were being studied due to short (n = 18) or tall stature (n = 3), otherwise healthy. The remainder were recruited as healthy control volunteers (n = 23). All boys were investigated over a 10 years period at Göteborg Pediatric Growth Research Centre at the Queen Silvia Children's Hospital in Göteborg, Sweden. The 44 boys had participated either in a cross-sectional study consisting of 36 boys at various pubertal stages or in a semi-longitudinal study of 8 boys followed-up with measurements twice (n = 1), three (n = 5) or four times (n = 1) or throughout pubertal development (n = 1).

Assent to inclusion in the study was obtained from each boy and informed consent from parents. The protocol was approved by the Ethics Committee of the Medical Faculty at the Sahlgrenska Academy at University of Gothenburg.

### Blood sampling and clinical protocol

A total of sixty-two 24-hour serum profiles were taken from the 44 boys. Serum estradiol concentrations in mid-puberty and late puberty have in part been reported previously for semi-longitudinally followed participants [14]. The 44 boys stayed in the hospital for at least 2 days for each serum profile. A heparinized needle was inserted during the first evening or morning. Blood for estradiol and testosterone measurements was taken at 1000, 1400, 1800, 2200, 0200 and 0600 h. After centrifugation, the serum samples were stored in -20 C until assayed. The storage time varied from 1 month up to 13 years (median 7 years) and all samples underwent a maximum of one freeze-thaw cycle before assay of estradiol.

During each visit to the clinic, height was measured by stadiometer, testicular volumes were determined by orchidometer [15] and bone age was assessed using the method of Tanner and Whitehouse (TW II) [16]. Based on the results from testosterone concentrations in relation to testicular volume [8], each boy's puberty was classified into five stages: pre (testis 1–2 mL), early (testis 3–6 mL), mid (testis 8–12 mL), late-1 (testis 15–25 mL, from 1 year before PHV up to 2 years after PHV, not reached final height) and late-2 (testis 15–25 mL, 2–3 years after PHV, reached final height). Further detailed information about the boys is described in a previous report [8].

### Estradiol determination

Serum 17β-estradiol concentrations were determined in duplicate by an assay which involves a diethyl ether extraction step prior to quantification by a modified commercial RIA (Spectria^® ^Estradiol RIA, Orion Diagnostica; Espoo, Finland). The extraction estradiol RIA has been meticulously validated for pediatric use [11,17]. The analytical detection limit for the extraction RIA was 4 pmol/L, calculated as the apparent concentration 3 SD from the counts at maximum binding, multiplied with the concentration factor obtained in the extraction step. The functional sensitivity was 6 pmol/L, defined as the lowest concentration that can be measured with an interassay CV of 20%. The recovery of estradiol using the extraction procedure was 91% [11] and all results have been corrected for the extraction loss. The cross-reactivity of the 17β-estradiol antiserum had been tested for 30 estradiol-related steroids. The cross-reactivity was below 1% for estrone, 16-oxoestradiol, estradiol-3-glucuronide, estriol, and 16-hydroxyestrone, below 0.1% for progesterone, corticosterone, 2-hydroxyestradiol, and below 0.001% for the rest of the related steroids. The intra-assay CV was 10–17% in the 5–37 pmol/L range, while the interassay CV was 19% at 6 pmol/L and below 14% for concentrations of 12 pmol/L and above. The extraction estradiol RIA has been shown to perform well in clinical practice for more than a decade at our laboratory and is an accredited assay by SWEDAC in Sweden, SS-EN ISO 15189 (no 1899).

For estimation of stability, three pooled samples from children at different serum estradiol levels had been thawed seven times to evaluate repeated thawing. As a check of long duration storage, aliquots of three serum pools (10, 20 and 600 pmol/L) were stored at -20 C and determination of estradiol concentrations have been done yearly for seven years. It was found that the estradiol concentrations were not affected by long term storage or affected by repeated freeze/thaw cycles.

### Statistical procedures

Non-parametric statistical methods were used for analyses. The Mann-Whitney test was used for comparison in estradiol concentrations between pre-, early and mid-pubertal stages and therefore boys followed with repeated estradiol measurements (n = 2) were excluded. The Wilcoxon signed rank sum test was used for analysis of diurnal variations and the progress of estradiol concentrations between mid, late-1 and late-2 pubertal stages as the progress was mainly based on serum from boys who had participated several times. In statistical analyses and figures, values of estradiol below the detection limits were set to the expected value of 2 pmol/L (the detection limit divided by 2). Mean estradiol levels were calculated as the mean value of six samples drawn over an interval of 24 hours. As the serum estradiol and testosterone levels were not normally distributed, linear regression analyses and 5^th ^and 95^th ^percentiles were performed on log-transformed data. A p-value of < 0.05 was considered significant.

## Results

### Serum estradiol levels and diurnal rhythm in boys

**Figure 1:**
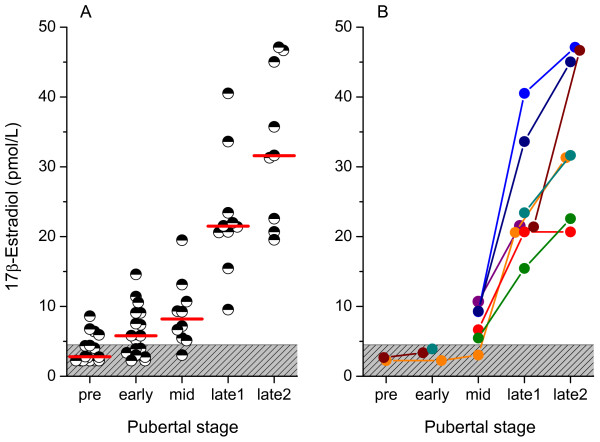
**Mean estradiol concentration during 24-hours secretion**. Concentrations below the detection limit (patterned area) during 24-hour sampling were set to the expected value of 2 pmol/L. A. Mean estradiol during 24 hours from 62 serum profiles. The bars show the median for the estradiol level in each pubertal group. B. Mean estradiol during 24 hours serum profiles in samples from 8 boys who have been followed-up with repeated estradiol measurements during pubertal development.

**Figure 2:**
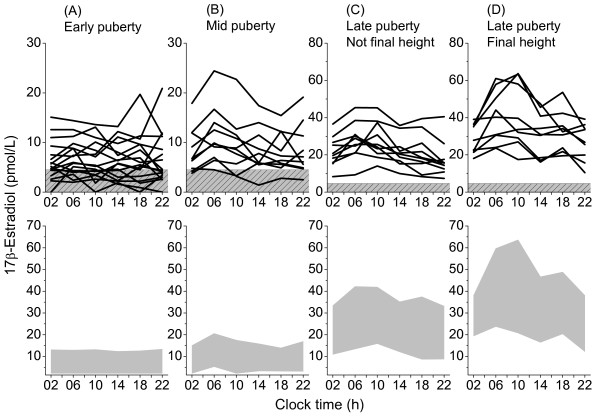
**Twenty-four hours secretion pattern of serum estradiol in healthy pubertal boys**. Upper panel: Individual 24-hour serum profiles of estradiol in 30 healthy boys. The patterned areas indicate values below the analytical detection limit. Lower panel: Normative data for the 24-hours secretion of serum estradiol. Shaded areas show 5–95 percentiles for each pubertal stage. Data is based on 17 boys in early puberty with testicular volume 3–6 mL (A), 10 boys in mid-puberty with testicular volume 8–12 mL (B), 10 boys in late-1 puberty, with testicular volume 15–25 mL, -1 to +2 years from peak height velocity (PHV), who have not reached final height (C) and 9 boys in late-2 puberty, with testicular volume 15–25 mL, +2 to +3 years from PHV and who have reached final height (D).

**Figure 3:**
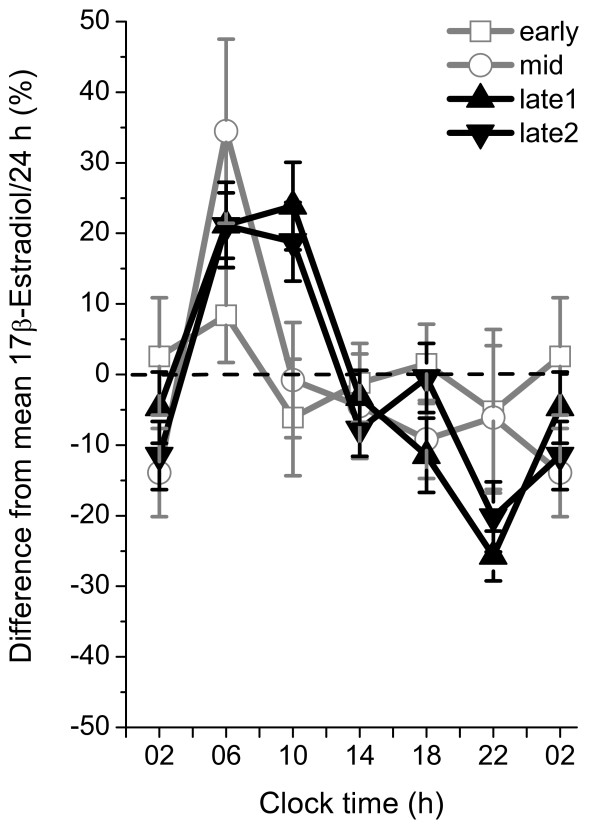
**Diurnal rhythm of serum estradiol during pubertal development in healthy boys**. For each study subject, the percentage difference from the mean value/24 h was calculated every four hours before dividing into pubertal groups. Data are shown as the mean value ± standard error of the mean for each pubertal group and clock time. Data is based on 17 boys in early puberty with testicular volume 3–6 mL, 10 boys in mid-puberty with testicular volume 8–12 mL, 10 boys in late puberty, with testicular volume 15–25 mL who have not reached final height and 9 boys in late puberty, with testicular volume 15–25 mL who have reached final height.

Serum estradiol levels increased progressively throughout male puberty (Figure 1). The increase was significant from prepuberty to early puberty (p < 0.01), from mid to late-1 puberty (p < 0.05) and from late-1 to late-2 puberty (p < 0.05). In prepubertal boys, the estradiol concentrations were barely detectable (Table 1). Six of the 16 prepubertal boys had no estradiol concentrations above the detection limit during the 24-hours sampling. In early puberty, 12 out of 17 boys had samples above the detection limit during the 24-hours sampling, however, no synchronized diurnal rhythm could be distinguished (Figure 2A). In mid-puberty, the majority of the boys showed serum estradiol concentrations above the detection limit. Seven out of ten boys displayed a diurnal rhythm, with low levels in the afternoon/evening and higher levels in the morning. Statistical analysis showed significantly higher estradiol concentrations at 0600 h compared to 1000 -1800 h and 0200 h (Table 1, Figure 2B). In late-1 puberty, a pronounced increase of estradiol levels took place as all boys had detectable concentrations at all sampling times and the diurnal rhythm was established with peak levels at 0600 to 1000 h, and 8 out of 10 had nadir at 2200 h. Boys in late puberty at final height had significantly higher estradiol concentrations compared to those in late puberty, who had not yet reached final height. Furthermore, their diurnal rhythm was characterized by peak levels at 0600 to 1000 h and nadir at 2200 or 0200 h (Table 1, Figures 2C, D). The relative diurnal rhythm was significantly higher in mid-puberty (48%), late-1 (50%) and late-2 (41%) puberty than early puberty (14%) when calculated as the percentage increase at 0600 to 1000 h from the nadir at 2200 to 0200 h, (Figure 3).

**Table 1:** Serum estradiol concentrations during a 24-hour profile in relation to pubertal stage in boys.

Pubertal stage	N	Chronological age (yr)	Bone age TWII (yr)	17β-estradiol (pmol/L) Median (95% confidence interval)					
				
				0200 h	0600 h	1000 h	1400 h	1800 h	2200 h
Pre	16	9.9 (7.2 to 12.9)	9.1 (6.2 to 11.6)	<4 (<4 – 5)	<4 (<4 – 9)	<4 (<4 – 6)	<4 (<4 – 5)	<4 (<4)	<4 (<4 – 5)
Early	17	12.4 (9.8 to 13.8)	11.7 (9.7 to 13.1)	5 (5 – 7)	6 (5 – 9)	5 (<4 – 8)	6 (<4 – 8)	6 (<4 – 10)	4 (<4 – 9)
Mid	10	13.5 (11.2 to 14.1)	13.0 (10.7 to 13.5)	7^e ^ (<4 – 12)	10^bd ^ (6 – 17)	9^e ^ (<4 – 13)	7^e ^ (5 – 14)	7^e ^ (5 – 12)	7 (5 – 15)
Late-1	10	15.2 (13.0 to 16.6)	15.2 (13.8 to 16.8)	20^ace ^ (15 – 30)	27^bcd ^ (21 – 39)	25^bcd ^ (19 – 38)	21^ace ^ (15 – 34)	18^ae ^ (9 – 35)	15^ae ^ (11 – 26)
Late-2	9	17.2 (16.6 to 18.6)	18.0 (15.4 to 18.0)	28^ae ^ (21 – 36)	40^cd ^ (24 – 58)	34^cd ^ (27 – 63)	31^ae ^ (17 – 46)	32 (22 – 42)	27^ae ^ (16 – 36)

P < 0.05 was considered to be significant

^*a *^Significantly lower than 1000

^*b *^Significantly higher than 1800

^*c *^Significantly higher than 2200

^*d*^Significantly higher than 0200

^*e *^Significantly lower than 0600

### Relation between estradiol and androgens

**Figure 4:**
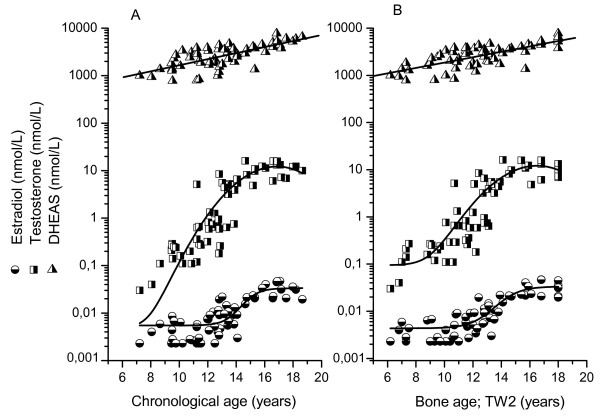
**Mean concentration during 24 hours of serum estradiol, testosterone and DHEAS in relation to chronological age and bone age (TW2) in healthy boys**. Estradiol and testosterone values below the detection limits are set to the expected values of 2 pmol/L and 0.02 nmol/L, respectively, before calculation of mean value.

The progress of serum estradiol, serum testosterone and serum DHEAS in relation to chronological age and bone age is presented in Figure 4. Although testosterone and DHEAS in boys shows more or less constantly increasing levels, the estradiol concentrations showed slow progress to a bone age of around 12 years. At bone age 14 years (that is pubertal stage late-1), all boys displayed estradiol levels over 20 pmol/L.

**Figure 5:**
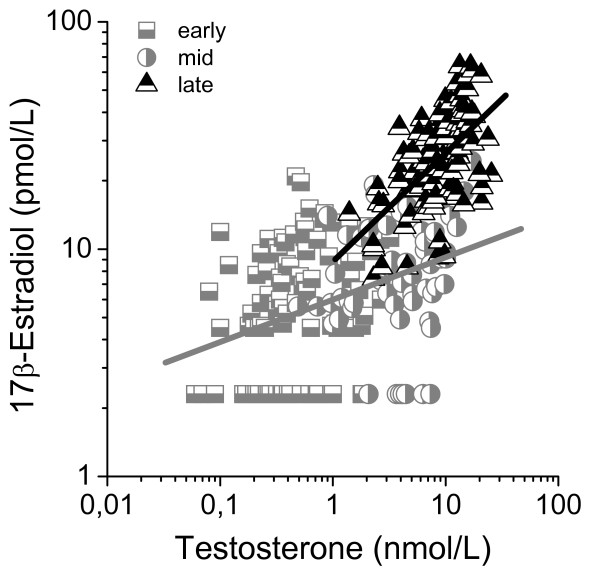
**Linear regression analysis between serum testosterone and serum estradiol during 24-hours sampling at different pubertal stages in healthy boys**. Boys followed up by repeated measurements were randomized to participate once in the analyses. Serum estradiol correlated significantly with serum testosterone: r = 0.38, p < 0.001 and r = 0.58, p < 0.001 for early to mid-puberty and late puberty, respectively. Estradiol values below the detection limit are set to the expected value of 2 pmol/L.

Serum testosterone levels associated strongly with total serum estradiol levels (R^2 ^= 0.56, p < 0.001). When dividing the group into different pubertal stages, the strongest correlation was found for late pubertal boys (r = 0.58, p < 0.001), where we also found the steepest regression line (Figure 5). The regression analysis for boys in early and mid-puberty showed similar regression lines and were therefore merged to one group in figure 5. Serum DHEAS concentrations correlated with serum estradiol concentrations (r = 0.61, p < 0.001), however, no correlation was found within each pubertal stage. Serum testosterone and DHEAS concentrations for this sample of boys have been published previously [8].

## Discussion

This is the most comprehensive study on the 24-hours estradiol levels and the diurnal variation of serum estradiol in healthy boys before puberty and during pubertal development. No diurnal rhythm was identified in pre- or early puberty, but it was discernable in mid-puberty and evident in late pubertal stages with peak values at 0600 to 1000 h and nadir at 2200 to 0200 h. Even if the diurnal rhythm of estradiol was not as marked as for testosterone in mid- and late pubertal boys [8], the intra-individual variability in estradiol from nadir to peak value was greater than the interassay CV for the extraction RIA. The present study is somewhat limited by the fact that low number of subjects were studied in each pubertal group.

The strengths of our estradiol assay is due to the fact that the RIA itself is sensitive as well as specific and that the extraction procedure removes interfering substances [11]. Furthermore, our assay has proved to determine results that, from the physician's point of view, are reliable and clinically useful to assess different endocrine conditions. E.g., it has in the present study and in previous studies [11,17] shown potential to distinguish prepubertal values from early pubertal values. It has shown ability to determine dose-response on breast development during hormone replacement therapy of girls [18] as well as during estrogen suppression therapy of girls with precocious puberty [11].

It is well established that direct immunoassays do not perform well at low estradiol concentrations, not even those with low analytical detection limit [10,11,13]. Therefore, with up-to-date knowledge, we are concerned that other previous studies on serum estradiol secretion in boys determined by direct immunoassays [6,19-23] have shown falsely high concentrations. In the present study, estradiol results for various pubertal stages in boys are comparable or somewhat higher compared with results determined with ultra-sensitive recombinant cell bioassays [24-26] and show similar or lower values compared to the few studies [27,28] that have used sensitive liquid chromatography-MS/MS.

The source of estradiol biosynthesis in the growing boy is complex and not fully understood. In adult men however, the major source of estradiol biosynthesis is aromatization of androgens in peripheral tissues e.g. muscles and adipocytes by endocrine or intracrine pathways or by sulfated precursors being hydrolysed and converted into estrogens [29-31]. Only 15–25% is synthesized in the testis. In our study it is obvious that a substantial increase in estradiol levels as well as a marked diurnal rhythm of estradiol does not appear until the end of puberty. During mid-puberty, the testosterone levels are not far from adult levels but the estradiol levels are still low. This is highlighted in figure 5 where testosterone concentrations (e.g. 10 nM testosterone) can be seen in both mid-puberty and late puberty resulting in 3 times higher levels of estradiol in late puberty than in mid-puberty. In the beginning of puberty, boys have a low aromatization capacity [32,33] which probably is due to a low capacity in the developing testis [34].

## Conclusion

With the use of an ultra-sensitive extraction RIA, designed for measuring low estradiol concentrations in serum, we have established reliable and clinically useful data from a group of healthy prepubertal and pubertal boys. Serum concentrations of estradiol increased from pre- to early puberty but then remained relatively constant until the marked increase between mid and late-1 puberty, and thereafter a slower increase until late-2 puberty. The diurnal rhythm of serum estradiol in boys was not apparent until mid- to late puberty. The slow progress of estradiol from early puberty to mid-puberty despite a marked increase in testosterone levels is most likely due to a limited aromatization capacity in the growing boy.

## Abbreviations

CV: coefficient of variation; DHEAS: dehydroepiandrosterone sulphate; MS: mass spectrometry; PHV: peak height velocity; RIA: radioimmunoassay; TW II: Tanner Whitehouse.

## Competing interests

The authors declare that they have no competing interests. The manuscript was partly funded by a grant by Pfizer.

## Authors' contributions

CAL participated in the design of the study, drafted the manuscript, carried out the radioimmunoassay and validation of the assay, performed analysis and interpretation of the data and the statistical analysis. EN conceived of the study, participated in its design, interpretation of the data and helped to draft the manuscript. Both authors read and approved the final manuscript.

## Pre-publication history

The pre-publication history for this paper can be accessed here:



## References

[B1] Hess RA, Bunick D, Lee KH, Bahr J, Taylor JA, Korach KS, Lubahn DB (1997). A role for oestrogens in the male reproductive system. Nature.

[B2] Cutler GB (1997). The role of estrogen in bone growth and maturation during childhood and adolescence. J Steroid Biochem Mol Biol.

[B3] de Ronde W, Pols HA, van Leeuwen JP, de Jong FH (2003). The importance of oestrogens in males. Clin Endocrinol (Oxf).

[B4] Hammoud AO, Gibson M, Peterson CM, Hamilton BD, Carrell DT (2006). Obesity and male reproductive potential. J Androl.

[B5] Tivesten A, Hulthe J, Wallenfeldt K, Wikstrand J, Ohlsson C, Fagerberg B (2006). Circulating estradiol is an independent predictor of progression of carotid artery intima-media thickness in middle-aged men. J Clin Endocrinol Metab.

[B6] Andersson AM, Juul A, Petersen JH, Muller J, Groome NP, Skakkebaek NE (1997). Serum inhibin B in healthy pubertal and adolescent boys: relation to age, stage of puberty, and follicle-stimulating hormone, luteinizing hormone, testosterone, and estradiol levels. J Clin Endocrinol Metab.

[B7] Mitamura R, Yano K, Suzuki N, Ito Y, Makita Y, Okuno A (1999). Diurnal rhythms of luteinizing hormone, follicle-stimulating hormone, and testosterone secretion before the onset of male puberty. J Clin Endocrinol Metab.

[B8] Ankarberg-Lindgren C, Norjavaara E (2004). Changes of diurnal rhythm and levels of total and free testosterone secretion from pre to late puberty in boys: testis size of 3 ml is a transition stage to puberty. Eur J Endocrinol.

[B9] Bay K, Andersson AM, Skakkebaek NE (2004). Estradiol levels in prepubertal boys and girls – analytical challenges. Int J Androl.

[B10] Stanczyk FZ, Cho MM, Endres DB, Morrison JL, Patel S, Paulson RJ (2003). Limitations of direct estradiol and testosterone immunoassay kits. Steroids.

[B11] Ankarberg-Lindgren C, Norjavaara E (2008). A purification step prior to commercial sensitive immunoassay is necessary to achieve clinical usefulness when quantifying serum 17beta-estradiol in prepubertal children. Eur J Endocrinol.

[B12] Nelson RE, Grebe SK, DJ OK, Singh RJ (2004). Liquid chromatography-tandem mass spectrometry assay for simultaneous measurement of estradiol and estrone in human plasma. Clin Chem.

[B13] Lee JS, Ettinger B, Stanczyk FZ, Vittinghoff E, Hanes V, Cauley J, Chandler W, Settlage J, Beattie M, Folkerd E (2006). Comparison of Methods to Measure Low Serum Estradiol Levels in Postmenopausal Women. J Clin Endocrinol Metab.

[B14] Albertsson-Wikland K, Rosberg S, Lannering B, Dunkel L, Selstam G, Norjavaara E (1997). Twenty-four-hour profiles of luteinizing hormone, follicle-stimulating hormone, testosterone, and estradiol levels: a semilongitudinal study throughout puberty in healthy boys. J Clin Endocrinol Metab.

[B15] Prader A (1966). Testicular size: assessment and clinical importance. Triangle.

[B16] Tanner J, Whitehouse R, Cameron N, Marshall W, Healy M, Goldstein H (1983). Assessment of skeletal maturity and prediction of adult height (TW2 method).

[B17] Norjavaara E, Ankarberg C, Albertsson-Wikland K (1996). Diurnal rhythm of 17 beta-estradiol secretion throughout pubertal development in healthy girls: evaluation by a sensitive radioimmunoassay. J Clin Endocrinol Metab.

[B18] Ankarberg-Lindgren C, Elfving M, Wikland KA, Norjavaara E (2001). Nocturnal application of transdermal estradiol patches produces levels of estradiol that mimic those seen at the onset of spontaneous puberty in girls. J Clin Endocrinol Metab.

[B19] Wu FC, Borrow SM, Nicol K, Elton R, Hunter WM (1989). Ontogeny of pulsatile gonadotrophin secretion and pituitary responsiveness in male puberty in man: a mixed longitudinal and cross-sectional study. J Endocrinol.

[B20] Gässler N, Peuschel T, Pankau R (2000). Pediatric reference values of estradiol, testosterone, lutropin, follitropin and prolactin. Clin Lab.

[B21] Ikegami S, Moriwake T, Tanaka H, Inoue M, Kubo T, Suzuki S, Kanzakili S, Seino Y (2001). An ultrasensitive assay revealed age-related changes in serum oestradiol at low concentrations in both sexes from infancy to puberty. Clin Endocrinol (Oxf).

[B22] Elmlinger MW, Kuhnel W, Ranke MB (2002). Reference ranges for serum concentrations of lutropin (LH), follitropin (FSH), estradiol (E2), prolactin, progesterone, sex hormone-binding globulin (SHBG), dehydroepiandrosterone sulfate (DHEAS), cortisol and ferritin in neonates, children and young adults. Clin Chem Lab Med.

[B23] Soldin OP, Hoffman EG, Waring MA, Soldin SJ (2005). Pediatric reference intervals for FSH, LH, estradiol, T3, free T3, cortisol, and growth hormone on the DPC IMMULITE 1000. Clin Chim Acta.

[B24] Klein KO, Martha PM, Blizzard RM, Herbst T, Rogol AD (1996). A longitudinal assessment of hormonal and physical alterations during normal puberty in boys. II. Estrogen levels as determined by an ultrasensitive bioassay. J Clin Endocrinol Metab.

[B25] Paris F, Servant N, Terouanne B, Balaguer P, Nicolas JC, Sultan C (2002). A new recombinant cell bioassay for ultrasensitive determination of serum estrogenic bioactivity in children. J Clin Endocrinol Metab.

[B26] Janfaza M, Sherman TI, Larmore KA, Brown-Dawson J, Klein KO (2006). Estradiol levels and secretory dynamics in normal girls and boys as determined by an ultrasensitive bioassay: a 10 year experience. J Pediatr Endocrinol Metab.

[B27] Kushnir MM, Rockwood AL, Bergquist J, Varshavsky M, Roberts WL, Yue B, Bunker AM, Meikle AW (2008). High-sensitivity tandem mass spectrometry assay for serum estrone and estradiol. American journal of clinical pathology.

[B28] Mauras N, Gonzalez de Pijem L, Hsiang HY, Desrosiers P, Rapaport R, Schwartz ID, Klein KO, Singh RJ, Miyamoto A, Bishop K (2008). Anastrozole increases predicted adult height of short adolescent males treated with growth hormone: a randomized, placebo-controlled, multicenter trial for one to three years. J Clin Endocrinol Metab.

[B29] Weinstein RL, Kelch RP, Jenner MR, Kaplan SL, Grumbach MM (1974). Secretion of unconjugated androgens and estrogens by the normal and abnormal human testis before and after human chorionic gonadotropin. The Journal of clinical investigation.

[B30] Reed MJ, Purohit A, Woo LW, Newman SP, Potter BV (2005). Steroid sulfatase: molecular biology, regulation, and inhibition. Endocr Rev.

[B31] Akingbemi BT (2005). Estrogen regulation of testicular function. Reprod Biol Endocrinol.

[B32] Dunkel L, Perheentupa J, Sorva R (1985). Single versus repeated dose human chorionic gonadotropin stimulation in the differential diagnosis of hypogonadotropic hypogonadism. J Clin Endocrinol Metab.

[B33] Cuttler L, Rosenfield RL, Ehrmann DA, Kreiter M, Burstein S, Cara JF, Levitsky LL (1993). Maturation of gonadotropin and sex steroid responses to gonadotropin-releasing hormone agonist in males. J Clin Endocrinol Metab.

[B34] Hess RA (2003). Estrogen in the adult male reproductive tract: a review. Reprod Biol Endocrinol.

